# Meta-analysis of the clinical behavior of posterior direct resin restorations: Low polymerization shrinkage resin in comparison to methacrylate composite resin

**DOI:** 10.1371/journal.pone.0191942

**Published:** 2018-02-21

**Authors:** Paula de Castro Kruly, Marcelo Giannini, Renata Corrêa Pascotto, Laíse Midori Tokubo, Uhana Seifert Guimarães Suga, Any de Castro Ruiz Marques, Raquel Sano Suga Terada

**Affiliations:** 1 Department of Dentistry, State University of Maringa, Maringa, Parana, Brazil; 2 Department of Restorative Dentistry, State University of Campinas, Piracicaba, São Paulo, Brazil; 3 Department of Pharmacology and Therapeutics, State University of Maringa, Maringa, Parana, Brazil; Helmholtz-Zentrum Dresden-Rossendorf, GERMANY

## Abstract

Polymerization shrinkage of resin composite can compromise the longevity of restorations. To minimize this problem, the monomeric composition of composites have been modified. The objective of this study was to conduct a meta-analysis to assess the clinical behavior of restorations performed with low polymerization shrinkage resin composite in comparison with traditional methacrylates-based resin composite. This systematic review was registered at Prospero data system (CRD42015023940). Studies were searched in the electronic databases PubMed, Web of Science, Scopus, Lilacs and EMBASE according to a predefined search strategy. The inclusion criteria were as follow: (1) randomized controlled clinical trials with at least six months of follow-up; (2) studies investigating composites with monomers designed to reduce polymerization shrinkage; (3) studies conducted with class I or II restorations in the permanent dentition; and (4) studies that assessed at least one of the following criteria: marginal integrity/adaptation, marginal discoloration, recurent caries, retention of composite restorations, and postoperative sensitivity. Two independent reviewers analyzed the articles to determine inclusion and risk of bias. The search conducted in the databases resulted in a total of 14,217 studies. After reviewing the references and citations, 21 articles remained. The longest clinical follow-up time was 60 months. The meta-analysis of the data in the included studies demonstrated that only one variable (marginal adaptation after 12 months) showed statistically significant outcomes, in which methacrylates-based composites presented significantly better results than resin composites containing modified monomers. The good level of the scientific evidence as well as the overall low risk of bias of the included studies indicate that composites with silorane, ormocer or bulk-fill type modified monomers have a clinical performance similar to conventional resin composites.

## Introduction

Over the past decades, resin composites came as a great promise to replace amalgam in dental restorations. Besides their low cost and esthetic appearance, composite restorations do not require extensive preparations, preserving dental structure, and present good clinical behavior in posterior teeth [1–3]. Literature reviews evaluating the longevity of conventional resin composite restorations in posterior teeth have demonstrated predictable outcomes [4–6].

Many of the most common clinical problems presented by posterior teeth restorations such as secondary caries, restoration fractures, marginal infiltration and marginal discoloration have been related to polymerization shrinkage stress [7]. To minimize these problems, restorations with conventional resin composites are made in increments, an effective but time consuming technique.

In order to minimize the polymerization shrinkage stress problem, recent changes in resin composites have focused on the polymer matrix [8]. As a result, new resin composites with modified monomers [9], such as the ormocer and silorane resins have been developed in attempt to reduce long-term clinical problems caused by polymerization shrinkage stress. Single-increment composites (bulk-fill resins) have also been developed to facilitate clinicians’ work, reduce working time and simplify the restorative procedure [10, 11]. Laboratory studies show that resin composites with modified monomers present less volumetric polymerization shrinkage than the methacrylate resins [12–16]. However, clinical follow-up studies conducted so far seem to indicate that these composites present similar clinical performance when compared to conventional resin composites [17–19].

Systematic reviews represent the highest source of scientific evidence and have become increasingly important in the decision making process of many health professionals in terms of the best treatments available [20]. Since no systematic review has been conducted to investigate the evidence regarding the clinical performance of resin materials with new monomeric compositions and modifications, this meta-analysis study was performed in order to improve the knowledge in that field. In other words, the characteristics of target composites of this study were: 1- not contain as main monomer the BIS-GMA or traditional di- or methacrylates and 2- new monomers and modified monomers-containing composites allow to increase the depth of cure, modify the incremental restorative technique, reduce the volumetric shrinkage and/or polymerization stress.

Therefore, the objective of this systematic review was to conduct a meta-analysis of the data available in selected studies to analyze the clinical behavior of restorations performed with low polymerization shrinkage resin composite in comparison with methacrylates-based resin composite. The tested null hypothesis was that restorations performed with low polymerization shrinkage composites would not show the same clinical performance as those performed with conventional methacrylates-based resin composites.

## Materials and methods

### Protocol and registration

This systematic review followed the recommendations established by the PRISMA (Preferred Reporting Items for Systematic Reviews and Meta-Analyses) protocol [21], and was registered at Prospero (International Register of Prospective Systematic Review) under the No. CRD42015023940.

### Eligibility criteria

The inclusion criteria were as follow: (1) randomized controlled clinical trials with at least 6 months follow-up time; (2) studies investigating composites with monomers designed to reduce polymerization shrinkage; (3) studies conducted with class I or II restorations in the permanent dentition; and (4) studies that assessed at least one of the following criteria: marginal integrity/adaptation, marginal discoloration, recurrent caries, retention of composite restorations, and postoperative sensitivity.

### Database

Study selection was conducted in the following electronic databases: PubMed, Web of Science, EMBASE, Scopus and Lilacs. No filter was used for specific languages. Searches were saved in RIS format to be opened in the EndNote reference management software.

### Search strategies

The following mesh terms with their respective entry terms were used: Composite Resins, Silorane Resins, Organically Modified Ceramics, Bulk-fill, Modified Monomers, Dimer Acid-based Monomers, Spiro-orthocarbonates, TCD-urethane, Modified Urethane Dimethacrylate Resin. The keyword "bulk-fill" is not included in PubMed’s "mesh terms" list, but it was employed to increase the scope of studies. The final search used in PubMed, Web of Science and Lilacs is presented in Table 1. For Scopus and EMBASE databases, the search was adapted to the format of these platforms.

**Table 1:** Search strategy used in Pubmed.

**P**	resin OR resins OR Composite Resins OR Resins, Composite OR composite resin OR resin composite OR resin restorations OR Composite Restorative Systems
**I**	silorane OR siloranes OR silorane resins OR resin, silorane OR resins, silorane OR silorane system adhesive OR adhesive, silorane system OR adhesives, silorane system OR Silorane System Adhesives OR System Adhesive, Silorane OR System Adhesives, Silorane OR silorane composite OR silorane based OR silorane-based OR silorane-based composite OR monomers modified OR bulk-filled OR bulk fill OR dimer acid-based monomers OR dimer acid monomers OR dimer-acid-based OR dimer acid based methacrylates OR dimer acid-based dimethacrylate OR dimer acid OR nano-dimer technology OR dimer acid-derived dimethacrylate OR spiro-orthocarbonates OR spiro orthocarbonate OR spiro orthocarbonates OR spiro ortho- carbonate OR Spiro Ortho Carbonate OR TCD-urethane OR TCD-urethane diacrylate OR TCD-urethane based monomers OR TCD-DI-HEA OR modified urethane dimethacrylate resin OR DX-511 OR urethane dimethacrylate-based monomer OR Organically Modified Ceramics OR Ceramic, Organically Modified OR Ceramics, Organically Modified OR Modified Ceramic, Organically OR Modified Ceramics, Organically OR Organically Modified Ceramic OR Ormocer OR Ormocers

### Study selection

With the assistance of EndNote software, two reviewers (PCK and LMT) evaluated independently the titles and abstracts of articles retrieved from the databases. Abstracts considered potentially eligible, as well as those which did not provide sufficient information on the eligibility criteria, were separated for full text evaluation. The two reviewers assessed independently the full texts to determine eligibility. In case of disagreement, which could not be resolved by consensus, a third reviewer (RSST) established the final decision. Afterwards, searches were performed on the reference list of selected articles, and authors were contacted when necessary.

### Risk of bias and quality of evidence

Risk bias analysis was performed with the tool available in the Cochrane Handbook [22]. To determine the quality of the evidence of the articles included in the review, a tool called GRADE [23] was also used.

### Data extraction

Two reviewers (PCK and LMT) conducted data extraction. General information such as: authors, year of publication, and geographic region of the first author, as well as the following specific characteristics were collected from each study: objective, place of research, number of centers involved in the study, patient recruitment period, type of material tested, inclusion and exclusion criteria, number of restorations performed and evaluated, type of restoration, duration of clinical follow-up, strategy used to evaluate restorations, criteria evaluated in each article, and authors’ conclusions.

### Data analysis

Data on the clinical performance of restorations conducted with composites containing new, modified monomers and methacrylate resin composites evaluated were: marginal integrity/adaptation, marginal discoloration, recurrent caries, retention of resin restorations, and postoperative sensitivity. The RevMan software was used to perform the meta-analysis and create the comparative tables for each clinical criterion, according to the different follow-up assessment periods.

## Results

**Fig 1:**
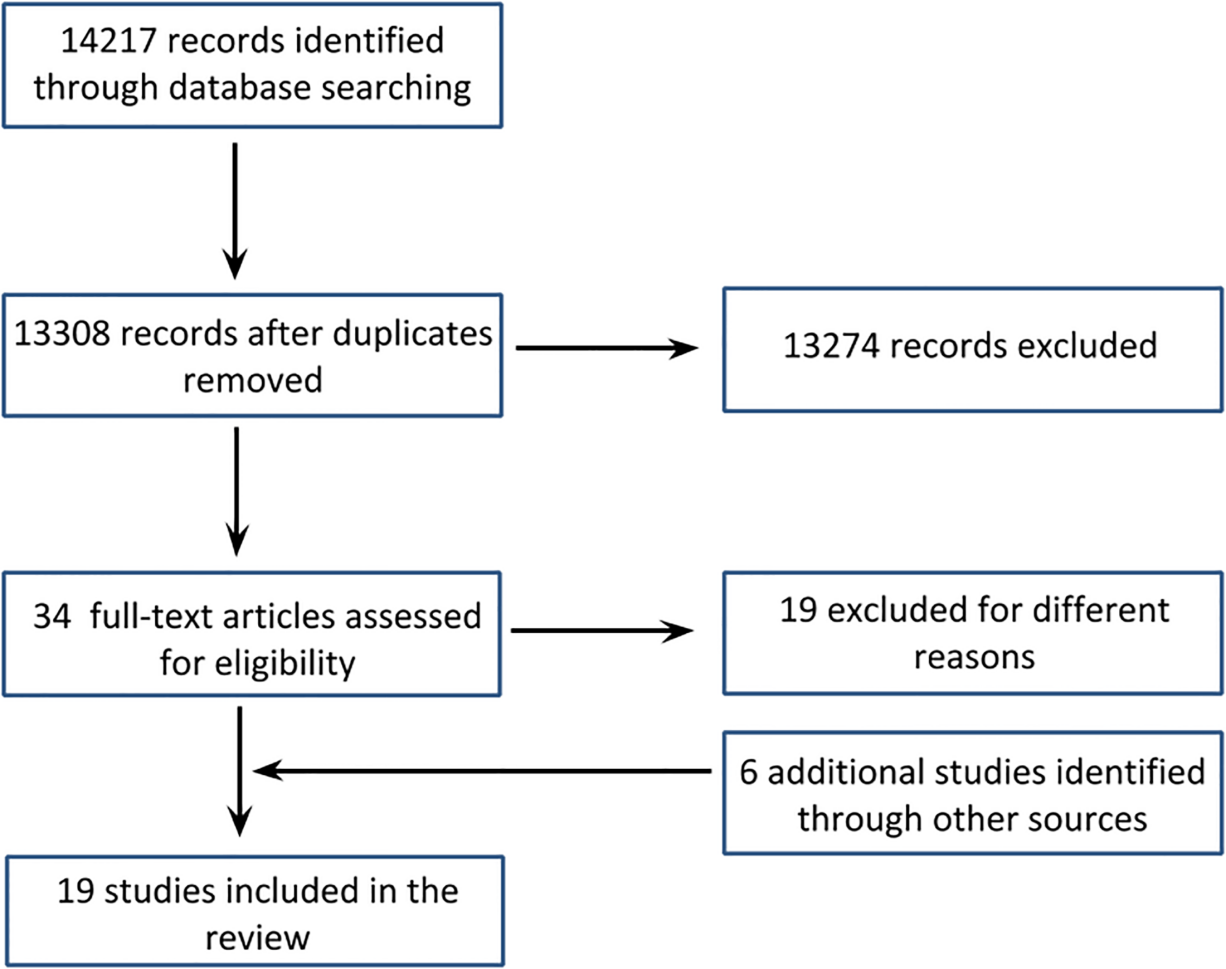
Flowchart showing the number of publications identified, retrieved, extracted, and included in the final analysis.

The search conducted in the databases resulted in a total of 14,217 studies, (13,308 after the removal of the duplicates). From these, 34 were selected for full text analysis, and 19 were excluded for different reasons: 1- no control group, 2- did not state whether groups were randomized, 3- evaluated indirect restorations, 4- in vitro studies, 5- the control group was not methacrylate-based composite, or 6- no clinical criterion was used. After reviewing the references and citations, from 15 selected articles, 6 references that did not appear in the search and which met the eligibility criteria were also included, totaling the 21 studies (Fig 1). One of the selected studies was not included in the meta-analysis due to lack of information in the results table [24].

The 21 articles included in the review and their characteristics and findings are presented in Tables 2 and 3.

**Table 2:** Characteristics of the studies included in the systematic review.

Study	Adhesive systems	Resins	Place of research	Restorations	Assessment criteria
Bayraktar, 2016 [17]	Single Bond Universal; AdheSE Bond; OptiBond All-In-One	Filtek Bulk-Fill Flowable Restorative; Tetric EvoCeram Bulk-Fill; Sonic Fill	Kirikkale, Turkey	50 (3 groups)	Modified USPHS
Gasparello, 2016 [25]	Filtek P90 System Adhesive	Filtek P90	Cascavel, Brazil	10	USPHS
Karaman, 2016 [26]	Adper Single Bond 2	x-tra base	Atakum, Turkey	47	Modified USPHS
Schmidt, 2015 [19]	Silorane System Adhesive	FiltekTM Silorane	Aarhus, Denmark	80	Own criteria
Attia, 2014 [27]	LS Low Shrinkage Adhesive	Filtek P90	Tanta, Egypt	15	Modified USPHS
Beck, 2014 [28]	Prime&Bond NT	Ceram X mono	Vienna, Austria	881	Modified USPHS
El-Eraky, 2014 [29]	LS Low Shrinkage Adhesive	Filtek P90	Egypt	10	Modified USPHS
Mahmoud, 2014 [25]	Filtek P90 System Adhesive	Filtek P90	Mansoura, Egypt	78	Modified USPHS
Santos, 2014 [30]	Silorane System Adhesive	Filtek LS	London, Canada	41	Modified USPHS
van Dijken, 2014 [31]	Xeno V	SDR	Umeå, Sweden	53	Modified USPHS
Walter, 2014 [32]	Filtek LS System Adhesive	Filtek LS Low Shrink Posterior Restorative	Chapel Hill, USA	41	Hickel et el.
Yazici, 2014 [33]	Filtek Silorane Adhesive	Filtek Silorane	Ankara, Turkey	28	Modified USPHS
Baracco, 2013 [34]	Filtek Silorane Restorative System	Filtek Silorane Restorative System	Madrid, Spain	25	Modified USPHS
Efes, 2013 [35]	Filtek Silorane System Adhesive	Filtek Silorane	Istanbul, Turkey	50	Modified USPHS
Gonçalves, 2013 [36]	Silorane System Adhesive	Filtek P90	Belo Horizonte, Brazil	50	Modified USPHS
Baracco, 2012 [37]	Filtek Silorane Restorative System	Filtek Silorane Restorative System	Madrid, Spain	25	Modified USPHS
Schmidt, 2011 [38]	Silorane System Adhesive	FiltekTM Silorane	Aarhus, Denmark	80	Own criteria
Bottenberg, 2009 [18]	Admira Bond; Etch & Prime 3.0	Admira; Definite	Brussels, Belgium	44/43 (2 groups)	Modified USPHS
van Dijken, 2009 [39]	Excite	InTen-S	Umeá, Sweden	53	Modified USPHS
Bottenberg, 2007 [40]	Admira Bond; Etch & Prime 3.0	Admira; Definite	Brussels, Belgium	44/43 (2 groups)	Modified USPHS
Efes, 2006 [41]	Admira Bond	Admira	Istanbul, Turkey	27	Modified USPHS

**Table 3:** Summary of findings.

**Low polymerization shrinkage restorations compared with methacrylate restorations for Clinical Behavior**			
**Patient or population: People with permanent posterior teeth** **Intervention: Low polymerization shrinkage restorations** **Comparison: Methacrylate restorations**			
**Outcomes**	**Relative effect** **(95% CI)**	**No of teeth** **(studies)**	**Quality of the evidence** (GRADE)*
**Marginal Adaptation** 12 months	**OR 1.77** (1.25 to 2.50)	2280 (18 studies)	⊕⊕⊕⊝ **moderate** [25–28,34,37, 38,40] ⊕⊕⊕⊕ **high** [17,29,30,32,33,35,41]
**Marginal Discoloration** 12 months	**OR 1.53** (0.98 to 2.41)	2082 (16 studies)	⊕⊕⊕⊝ **moderate** [25–28,34,37,40] ⊕⊕⊕⊕ **high**[17,29,30,33,35,41]
**Secondary Caries** 12 months	**OR 1.51** (0.64 to 3.57)	2087 (16 studies)	⊕⊕⊕⊝ **moderate** [25–28,34,37] ⊕⊕⊕⊕ **high** [17,29,30,32,33,35,39,41]
**Retention** 12 months	**OR 0.83** (0.33 to 2.09)	1834 (13 studies)	⊕⊕⊕⊝ **moderate** [25–28,34,37] ⊕⊕⊕⊕ **high**[17,32,33,35,41]
**Postoperative sensitivity** 12 months	**OR 1.65** (0.71 to 3.81)	970 (13 studies)	⊕⊕⊕⊝ **moderate** [25,33,34,36,38] ⊕⊕⊕⊕ **high**[17,29,31,32,37]

* GRADE Working Group grades of evidence:

**High quality:** Further research is very unlikely to change our confidence in the estimate of effect.

**Moderate quality:** Further research is likely to have an important impact on our confidence in the estimate of effect and may change the estimate.

**Low quality:** Further research is very likely to have an important impact on our confidence in the estimate of effect and is likely to change the estimate.

**Very low quality:** We are very uncertain about the estimate.

**Fig 2:**
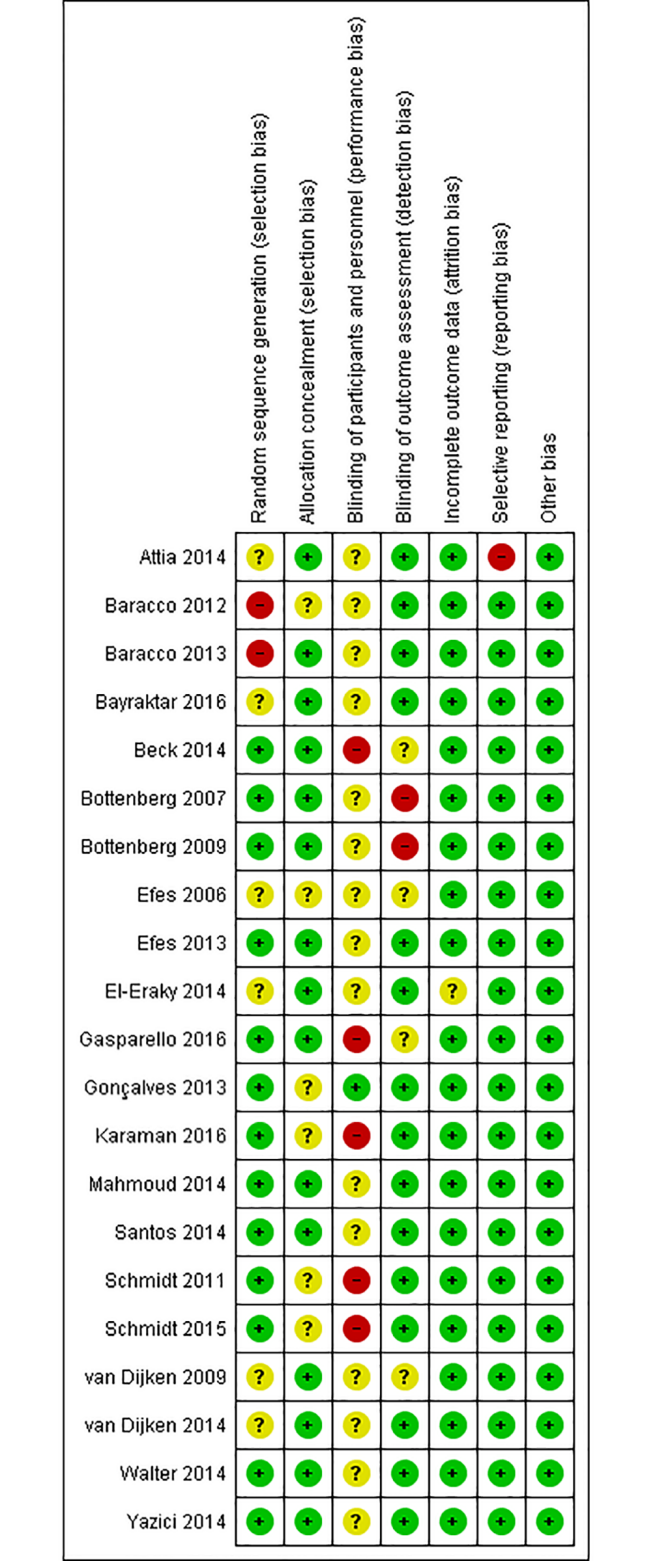
Risk of bias of selected studies.

Risk of bias of each selected study is illustrated in Fig 2. Green circles represent low risk of bias, red circles depict high risk of bias, and yellow circles indicate unclear risk of bias.

**Fig 3:**
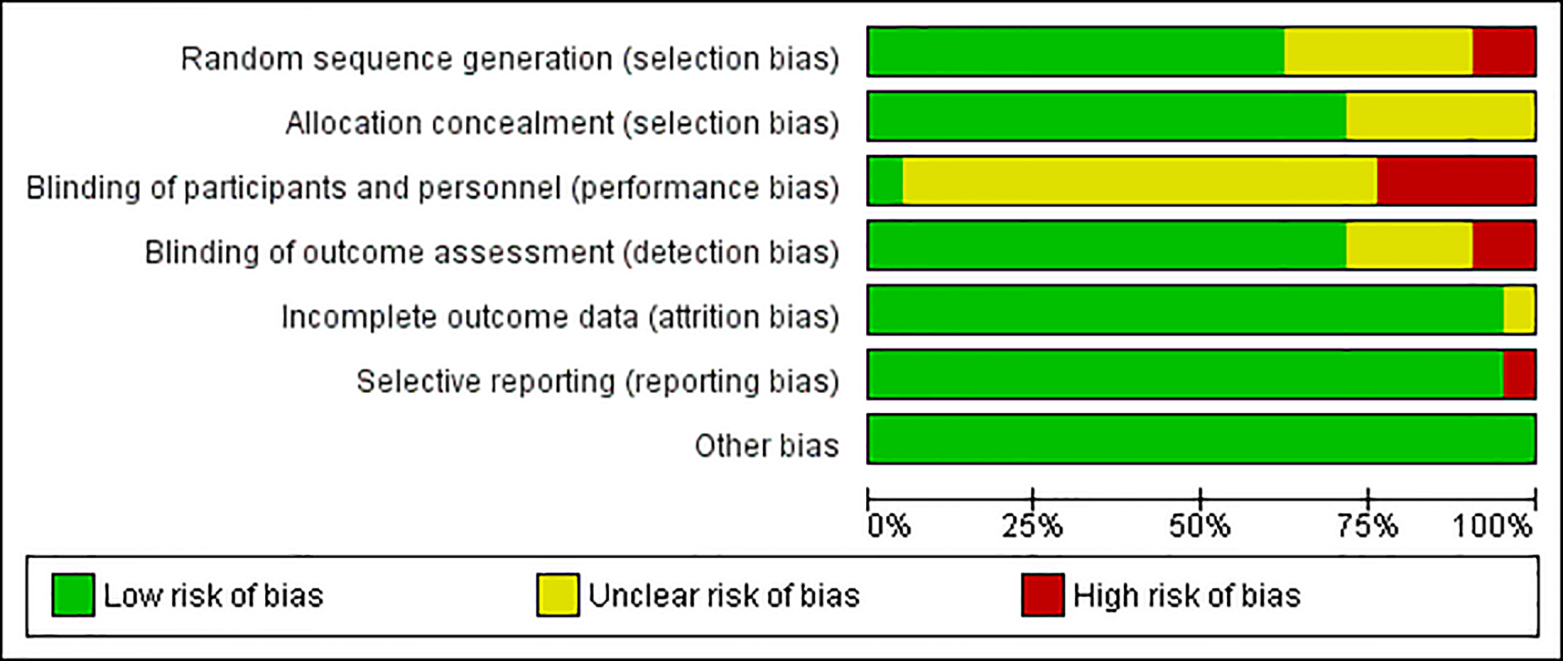
Graphic representation of risk of bias of the selected studies.

Fig 3 is a graphic illustration of the different risk of bias of all studies included in the review, indicating an overall low risk of bias.

From the 21 studies, 4 compared ormocer, 13 silorane, 3 bulk-fill and 1, InTen-S resin composites with conventional composites. None of the studies individually showed a significant difference between the tested materials for any of the analyzed variables, except for one study, in which silorane composite presented inferior marginal integrity results in comparison with conventional composite after a follow-up of 18 months [36]. All studies demonstrated that composites with new and modified monomers presented similar clinical results when compared to conventional composites.

### Marginal adaptation

**Fig 4:**
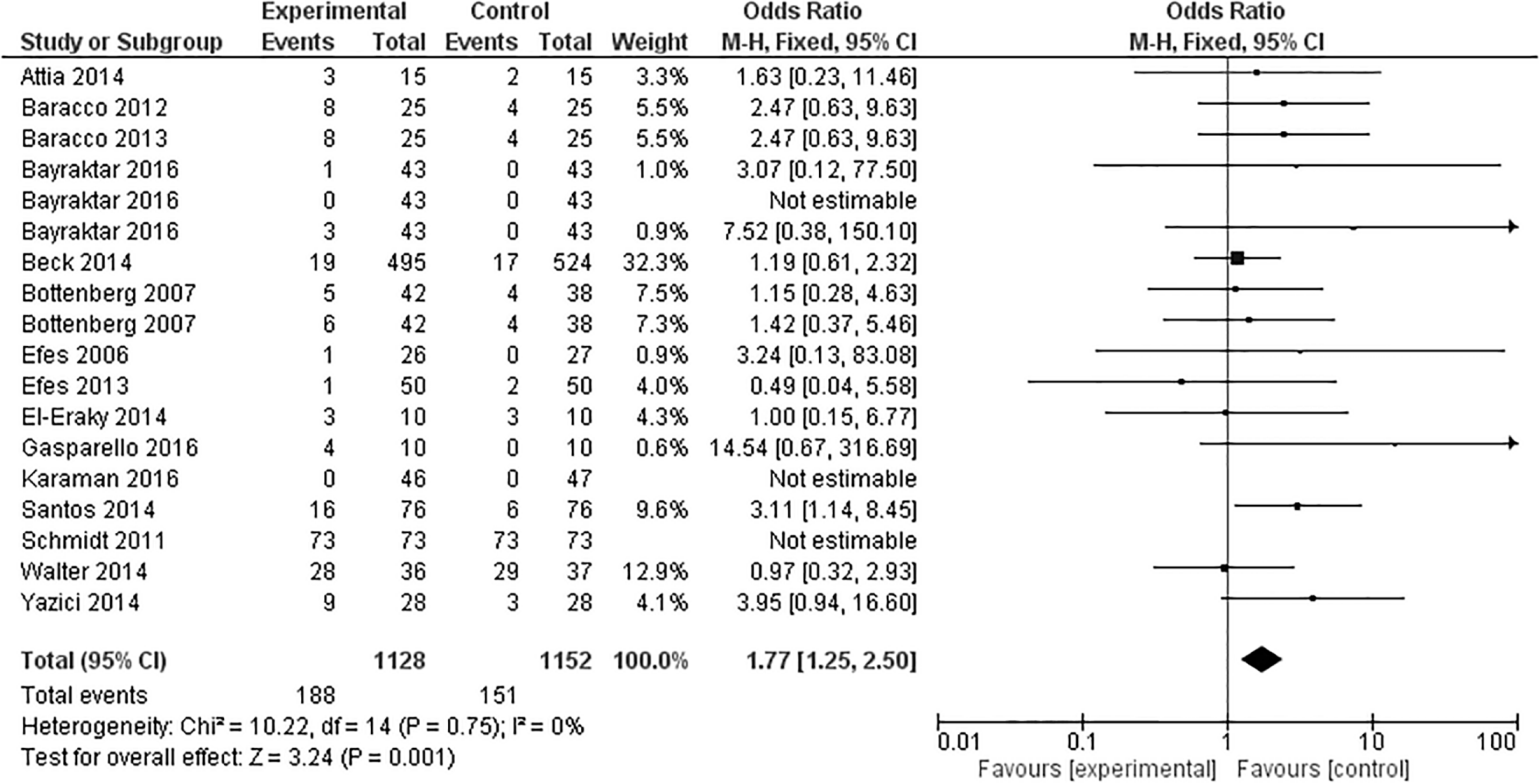
Marginal adaptation at the 12-month clinical follow-up examination.

Fifteen studies evaluated marginal adaptation after 12 months, with one study [17] presenting three experimental groups, and another [40] two experimental groups, totaling 18 experimental groups (Fig 4). Among the 18 groups, 12 presented results that favored the control group (conventional composite), [19,25,27,28,30,33,34,37,40,41] two that favored the experimental group (resins with modified monomers) [32,35], one that did not favor any of the groups, since results were similar for both groups [29], two presented no marginal adaptation alterations in both groups [17,26], and one study reported that all restorations in both groups demonstrated some sort of marginal adaptation alteration [38]. Meta-analysis demonstrated that at the 12-month follow-up assessment, the overall effect of methacrylates-based composite resins was significantly better than the ormocer, silorane and bulk-fill composites (p = 0.001).

Only two studies, both of which testing silorane composites, evaluated marginal adaptation after 18 months [29,36]. From these, one favored the experimental group [29] while the other the control group [36]. Although the result that favored the experimental group presented greater importance in the meta-analysis, the overall effect was not statistically significant.

**Fig 5:**
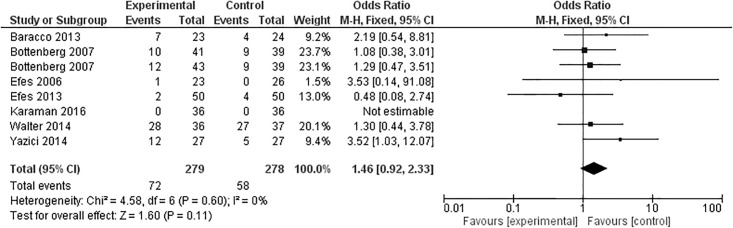
Marginal adaptation at the 24-month clinical follow-up examination.

Seven articles evaluated marginal adaptation after 24 months, one of them [40] with two experimental groups, totaling eight groups (Fig 5). Although six of these studies favored the control group [32–34,40,41], the overall effect was not statistically significant in the meta-analysis (p = 0.11).

Five articles evaluated marginal adaptation after 36 months, one of them [40] with two experimental groups. Among the six groups tested, two favored the control group [33,40], one favored the experimental group [31], one presented the same number of restorations with marginal adaptation alterations in both groups [32], and one presented no marginal adaptation alterations in both groups [26]. Meta-analysis demonstrated that overall effect was not statistically significant.

Three articles evaluated marginal adaptation after 60 months; one [18] with two experimental groups. From the four groups tested, two presented alterations in marginal adaptation in all restorations [18,19], and two favored the experimental group [18,39], but again the meta-analysis showed that the overall effect was not statistically significant.

### Secondary caries

**Fig 6:**
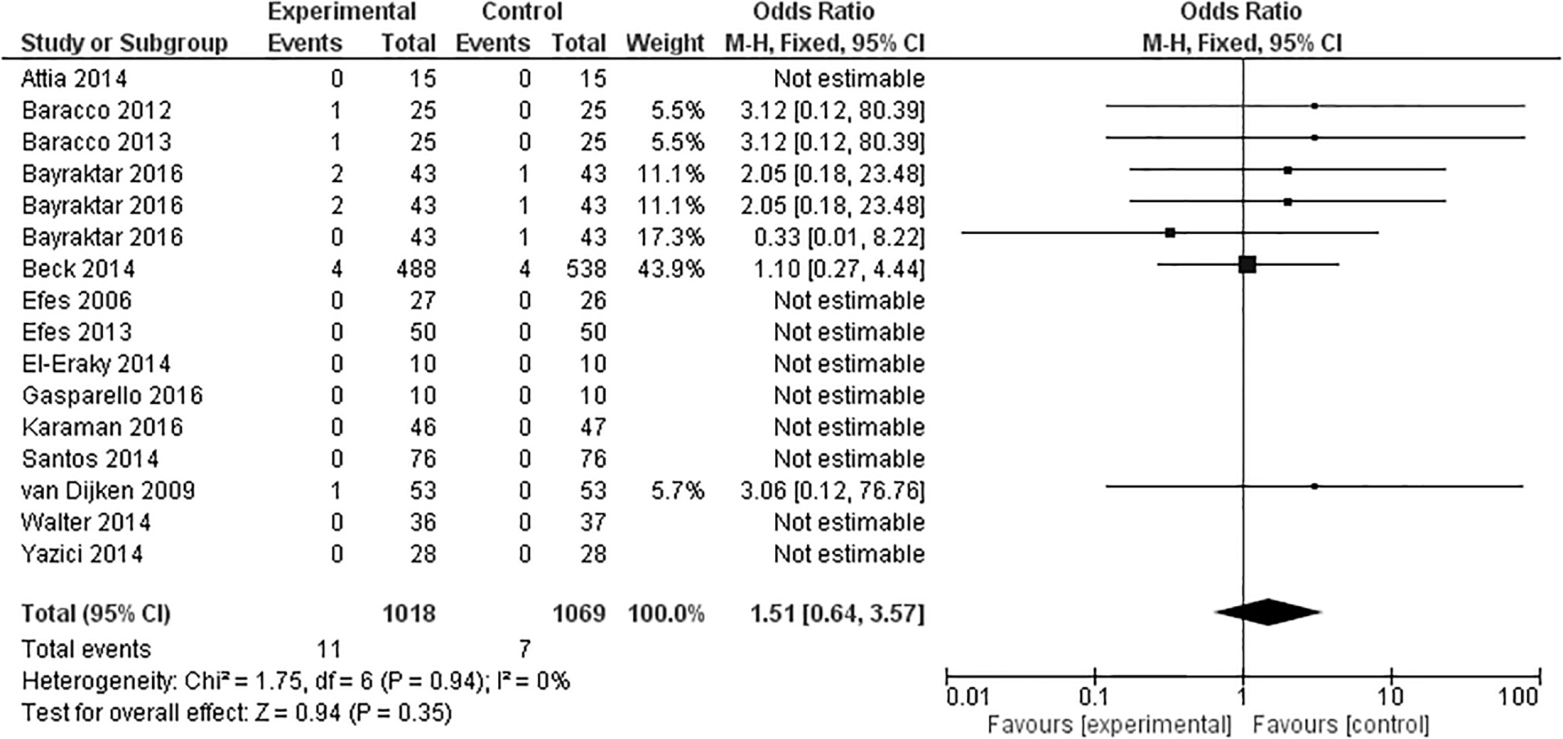
Secondary caries at 12-month clinical follow-up examination.

Fourteen articles evaluated the presence of secondary caries in the restorations after 12 months, one [17] with three experimental groups, totaling 16 groups (Fig 6). Nine studies demonstrated no secondary caries, neither in the experimental group nor in the control group [25–27,30,32,33,35,41]. One study presented results favoring the experimental group [17], while the other six favored the control group [17,28,34,37,39]. Meta-analysis demonstrated that the overall effect was not statistically significant (p = 0.38).

After 24 months, seven articles evaluated secondary caries [26,32–35,39,41], but none reported the presence of secondary caries, and meta-analysis was not conducted.

### Marginal discoloration

**Fig 7:**
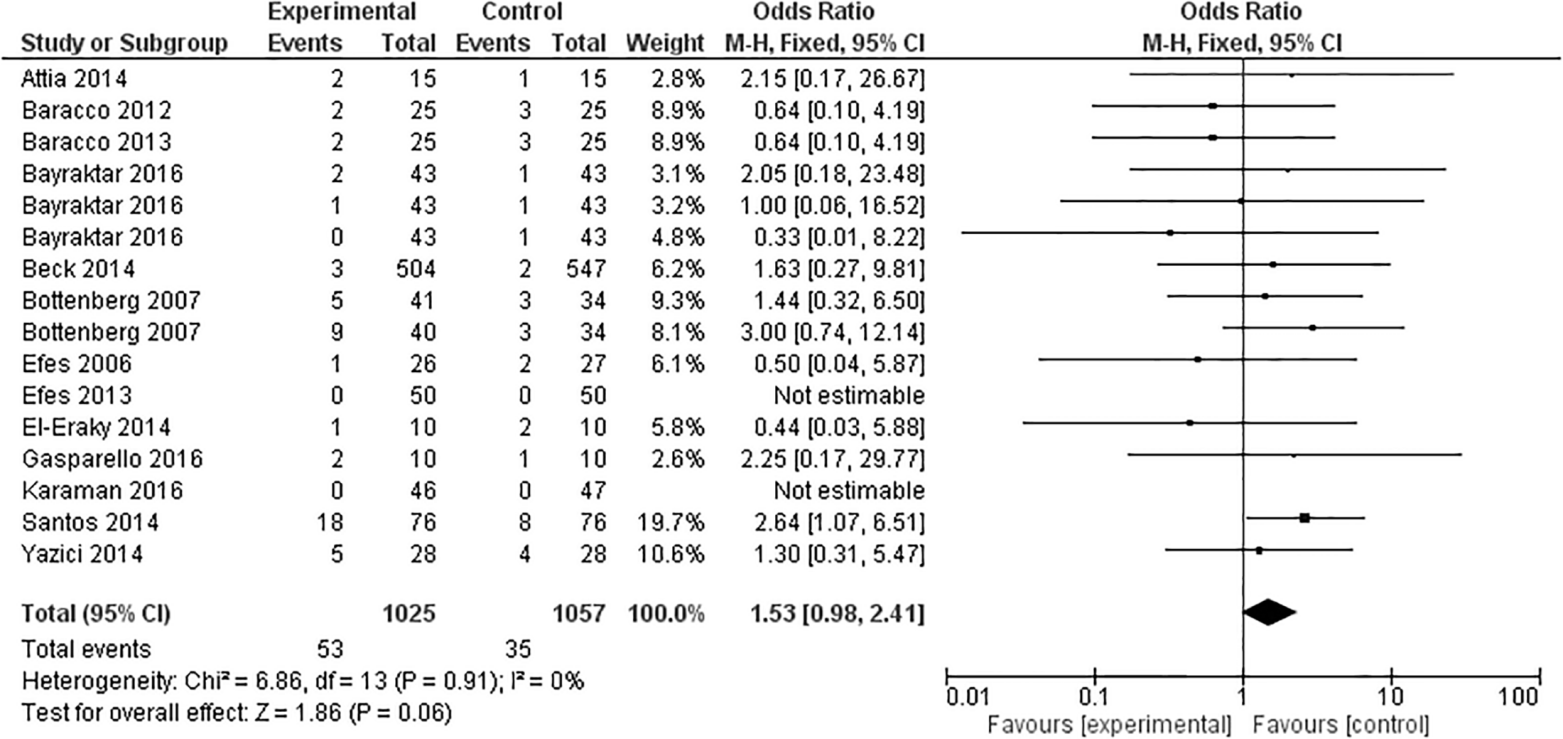
Marginal discoloration at the 12-month clinical follow-up examination.

Marginal discoloration was analyzed in thirteen articles after 12 months, with a total of 16 experimental groups being evaluated (Fig 7). One study [17] presented three experimental groups, and another [40] two experimental groups. From the 16 groups, eight favored the control group [17,25,27,28,30,34,40], five favored the experimental group [17,29,34,37,41], and one presented the same results for both groups [17]. Two studies presented no marginal discoloration in both groups [26,35]. Meta-analysis demonstrated that the overall effect was not statistically significant (p = 0.06).

Only two articles, both of which testing silorane resins, evaluated marginal discoloration after 18 months. Of these, one favored the experimental group [29], while the other the control group [36], with the meta-analysis showing no differences in the overall effect.

**Fig 8:**
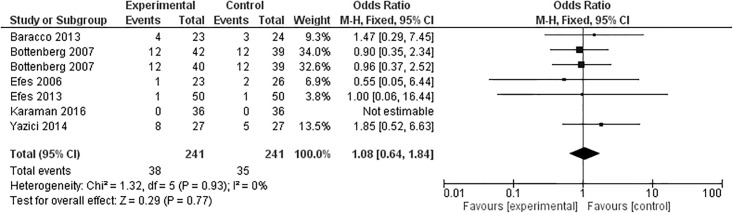
Marginal discoloration at the 24-month clinical follow-up examination.

Six articles evaluated marginal discoloration after 24 months; one study with two experimental groups [40], totaling seven groups (Fig 8). From these groups, three favored the experimental group [39,41], two favored the control group [33,34], one presented similar results for both groups [35], and one demonstrated no marginal discoloration in both groups [26]. Meta-analysis demonstrated that the overall effect was not statistically significant (p = 0.77).

Two articles [18,39], one with two experimental groups [18] evaluated marginal discoloration after 60 months. Two favored the experimental group, while the other presented an outcome that did not favor either group. Again, meta-analysis demonstrated no differences in the overall effect.

### Retention

**Fig 9:**
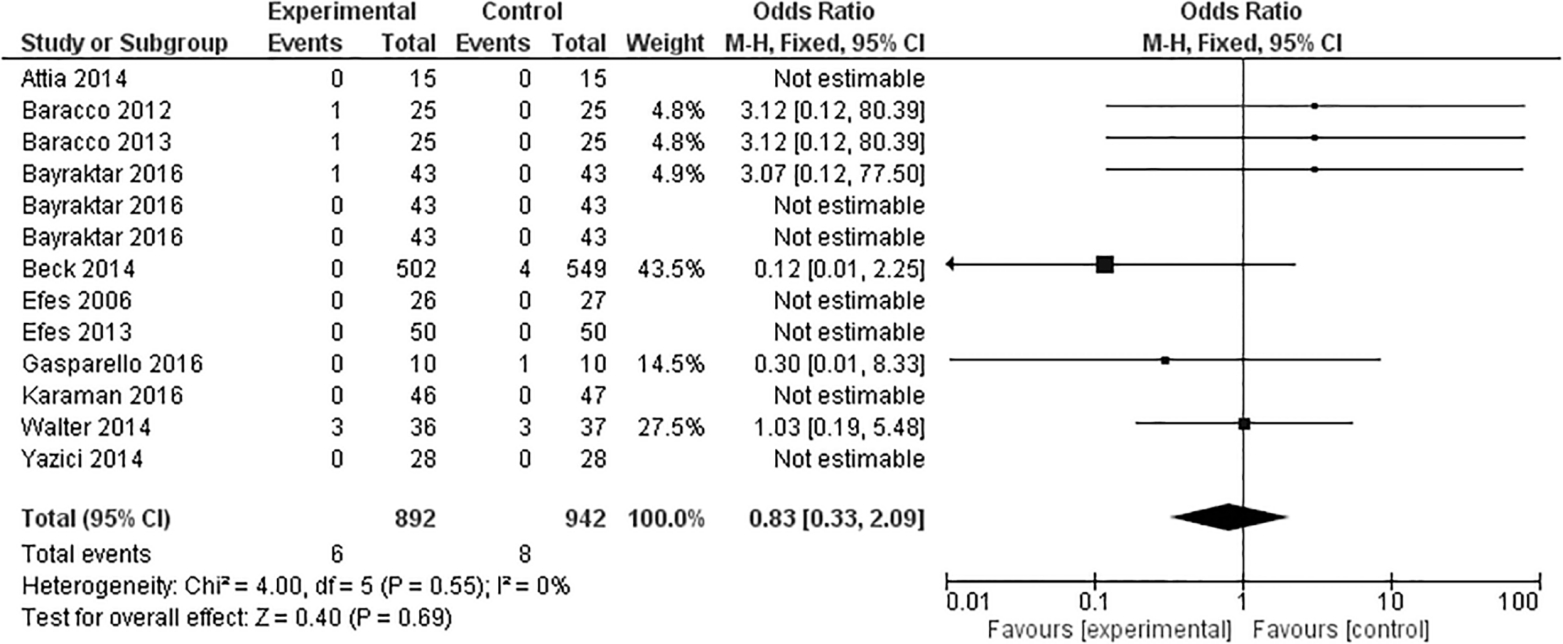
Retention at the 12-month clinical follow-up examination.

Eleven articles evaluated retention of composite restorations after 12 months; one [17] with three experimental groups (Fig 9). From the 13 groups analyzed, seven presented no loss of retention in either group [17,26,27,33,34,41], three favored the control group [17,34,37], two favored the experimental group [25,28], and one presented an outcome that did not favor any of the groups [32]. However, the meta-analysis of the studies indicated no statistically significant differences in the overall effect (p = 0.69).

Only six articles assessed restoration retention after 24 months, four of which did not show loss of retention in either group [26,33–35], while the other two favored the control group [32,41], with no differences in the overall effect.

### Postoperative sensitivity

Ten articles evaluated postoperative sensitivity after 12 months; one with two experimental groups [40], and another with three experimental groups [17], totaling 13 experimental groups. From these groups, nine demonstrated no postoperative sensitivity in any of the groups [17,25,30,33–35,37,41], three favored the control group [17,40], and one favored the experimental group [32]. Meta-analysis demonstrated no statistically significant differences in the overall effect.

Six articles evaluated postoperative sensitivity after 24 months; one with two experimental groups [40], totaling seven experimental groups. Four groups showed no postoperative sensitivity in any of the groups [33–35,41], while the other three favored the control group [32,40]. However, meta-analysis demonstrated no statistically significant differences in the overall effect.

## Discussion

This systematic review included 21 studies that compared the clinical performance of four different types of composites (ormocer, silorane, bulk-fill and InTen-S) with methacrylate resin composites. The evidence that emerged from the randomized controlled clinical trials included in this review indicates that the clinical behavior of low polymerization shrinkage composite resins in posterior class I and II restorations in the permanent dentition is similar to that of conventional methacrylates-based. Therefore, the null hypothesis must be rejected.

Because of the nature of the included studies, the quality of the evidence was considered good, and the risk of bias was low. Blinding of participants and personnel involved in the study (performance bias) was the most common problem in the selected studies, resulting in the largest number of unclear and high risk of bias. This is a problem difficult to circumvent, since omitting technical and product information from both patients and professionals is clinically unfeasible. Each material has specific characteristics and must follow recommendations concerning their insertion.

In the selected studies, restorations performed with conventional, silorane, and ormocer composites were performed incrementally, a technique indicated to overcome the problem of polymerization shrinkage and cuspal deflection [42]. Restorations conducted with bulk-fill resin composites were performed according to the technique recommended by manufacturers. In the case of flowable bulk-fill composites, increments of 4 to 5 millimeters were finished with conventional resin composite in the last 2 millimeters occlusally, while regular-consistency bulk-fill composites were performed in a single increment [17]. According to the manufacturers and laboratory studies [12,43], bulk-fill resin composites can be used in thick increments because they present low polymerization shrinkage and stress-relieving monomers.

This meta-analysis showed that the overall effect of the marginal adaptation of conventional restorations after a 12-month follow-up was better than that of composites with new and modified monomers, while for longer follow-up times no differences was observed between them. Several factors related to the restorative procedures and the characteristics of composites may influence marginal adaptation, as well as the operator. According to some authors, the type of resin composite and its viscosity influence the gap formation between the tooth and the restoration [44,45]. Regarding the restorative steps, the finishing and polishing procedures [46], as well as the insertion technique also influence marginal adaptation [47]. In all included studies, restorations underwent polishing procedures, but the direction of polishing, which is an important factor in marginal adaptation [46], was not disclosed.

Results of the 12-month follow-up evaluations showed that most restorations, regardless of the restorative material, did not present secondary caries. In those cases where lesions were present, the studies found no significant differences between experimental and control groups. The development of secondary caries lesions around the restoration is one of the main causes of resin composite restoration failures and the mechanism to control or avoid caries around restorations still under discussion [2,48]. Although some studies have suggested that secondary caries lesions may be influenced by the type of restorative material used [48], individual comportamental factors may also be involved in the development of these lesions. Moreover, the type of cavity and the location of the restoration may also influence the appearance of secondary caries lesions, which would characterize this particular condition as being multifactorial [48].

Several of the included studies evaluating marginal discoloration at the 12-month follow-up examination showed compromised restorations. Santos (2014) [30] reported that the worst marginal discoloration values were found in the experimental group using silorane resin composite. Marginal discoloration can be related to the nature of the adhesive system used in restorations [36,37]. Once again individual factors such as smoking and drinking (tea, coffee and wine) [49], as well as the placement of excessive amount of restorative material (“flash”), poorly marginal adaptation, and "gaps" can all contribute to increased marginal discoloration [37]. All restorations in the included studies were finished and polished, but patient’s habits regarding the intake of heavily pigmented drinks and smoking were not reported in the studies, clouding the assessment. However, no relationship between discoloration and caries were noted, and marginal discoloration of the restorations could often be solved with re-polishing [49,50].

Restoration retention is one of the main factors directly related to polymerization shrinkage of resin composites [32]. The volumetric shrinkage of the resin composite that is bonded to the dental wall of cavity preparation generates stresses that can result in loss of marginal adaptation and loss of retention [51]. In the 12-month follow-up evaluation, only four groups showed loss of retention in some restorations of experimental group, which did not seem to compromise the predictability of the restorative treatment.

Postoperative sensitivity may also occur as a consequence of polymerization shrinkage stress of resin composites [52,53]. Sensitivity is also often attributed to the infiltration of bacteria and other irritants along restoration margins into the pulp [49]. In both the 12-month and 24-month follow-up evaluations, most studies reported no clinical case with postoperative sensitivity. Among the studies that showed the presence of postoperative sensitivity, Bottenberg et al. (2007) [40] was the one that presented the worst results. In that study, deep cavities were lined with glass ionomer cement (Ketac Bond, 3M ESPE Seefeld, Germany), and polymerization time followed the manufacturer’s recommendations (40 to 60 seconds). Other studies that evaluated postoperative sensitivity after 12 months also reported that each increment was light activated for 40 seconds, but no case of sensitivity was reported [25,33,35,41].

The evidence that emerged from this systematic review was somewhat surprising. Due to the large investment in innovation and technology on the development of new restorative materials with monomeric modifications, it would be expected that evidence emerging from the recent literature would demonstrate some clinical superiority and advantages of the new resin composites compared to the conventional methacrylate-based composites. The criteria used to compare the clinical behavior of the different resin composites (marginal adaptation, secondary caries, marginal discoloration, retention and postoperative sensitivity) were chosen because these are related to polymerization shrinkage [12,44,45,50,54]. However, the actual polymerization shrinkage effects on the clinical behavior of the composite restorations needs to be considered carefully.

A recent study by Ferracane and Hilton (2016) [50] addressed the effect of polymerization shrinkage and stress on the clinical behavior of restorations. According to the authors, there is no conclusive evidence indicating that polymerization shrinkage may decrease the longevity of restorations, as its effects cannot be clearly distinguished from inadequate adhesion. On the other hand, reduced polymerization shrinkage by itself may not necessarily reduce stresses at the resin-tooth interface [37], and it does not seem to have any clinical significance [20]. Thus, it can not be said that the less polymerization shrinkage offered by composites with modified monomers will have a superior clinical behavior over conventional composites.

Successful restorations do not depend only on polymerization shrinkage, and several other aspects may influence longevity. While the physical and mechanical properties of materials are important, they cannot be solely responsible for the clinical performance of restorations. Operator’s experience, the location and conditions under which the treatment is performed, as well as the morphological characteristics of teeth, presence of contact points, occlusion, parafunctional habits, occlusal loading, and salivary composition also play a part in the final result [40].

Although the clinical behaviour of restorative composites with new and modified monomers has been shown to be predictable, they did not exhibit superior clinical longevity or performance than restorations conducted with conventional composites. Thus, clinicians should be cautious before deciding to change their restorative material and technique in posterior teeth. Although laboratory studies have shown that the new composition of resins can reduce the effects of polymerization shrinkage, aspects such as cost-benefit, and clinicians’ experience and ability need to te taken into consideration in clinical practice. New composites are more costly than conventional methacrylates-based composites, and some brands require more dexterity to achieve good sculpture. The benefits of the most recently new and modified monomer composites in the market, particularly bulk-fill composites, are apparently restricted to the shorter placement and light activation time. Interestingly, in the selected studies, the ormocer and silorane composites were placed incrementally, following the same technique used for conventional resin composites. In the case of bulk-fill composites, particularly those inserted in only one increment, it seems prudent that clinicians use them with caution, while no further studies confirming their clinical advantages are available [50]. As a recent meta-analysis concluded, there is still a big need for long-term clinical studies [55]. The CONSORT 2010 Statement [56] provides guidance for reporting randomised controlled trials and should be used. Furthermore, it is important define criteria to allow the effective evaluation of the resin effects [55] and the recommendations for conducting controlled clinical studies of dental restorative materials propose by Hickel et al. [57] provided relevant clinical evaluation parameters.

## Conclusions

The scientific evidence that emerged from this review of randomized controlled clinical trials indicates that restorations conducted with low polymerization shrinkage composites, such as silorane, ormocer and bulk-fill type showed clinical performance similar to restorations with conventional resin composites. Other aspects related to the long-term success of restorations need to be further investigated in order to better ascertain if any real advantage exist in the use of composites with new and modified monomers. The quality of the evidence of the included studies was considered good, and the risk of bias was low. However, the use of a guidance for reporting future randomised controlled trials and criteria to allow the effective evaluation of the resin effects are strongly recommended.

## Supporting information

S1 Table PRISMA checklist. (DOC)
